# Three-dimensional reconstruction of highly complex microscopic samples using scanning electron microscopy and optical flow estimation

**DOI:** 10.1371/journal.pone.0175078

**Published:** 2017-04-06

**Authors:** Ahmadreza Baghaie, Ahmad Pahlavan Tafti, Heather A. Owen, Roshan M. D’Souza, Zeyun Yu

**Affiliations:** 1 Department of Electrical Engineering, University of Wisconsin-Milwaukee, Milwaukee, WI, United States of America; 2 Biomedical Informatics Research Center, Marshfield Clinic Research Foundation, Marshfield, WI, United States of America; 3 Department of Biological Sciences, University of Wisconsin-Milwaukee, Milwaukee, WI, United States of America; 4 Department of Mechanical Engineering, University of Wisconsin-Milwaukee, Milwaukee, WI, United States of America; 5 Departments of Electrical Engineering and Computer Science, University of Wisconsin-Milwaukee, Milwaukee, WI, United States of America; Universidade de Mogi das Cruzes, BRAZIL

## Abstract

Scanning Electron Microscope (SEM) as one of the major research and industrial equipment for imaging of micro-scale samples and surfaces has gained extensive attention from its emerge. However, the acquired micrographs still remain two-dimensional (2D). In the current work a novel and highly accurate approach is proposed to recover the hidden third-dimension by use of multi-view image acquisition of the microscopic samples combined with pre/post-processing steps including sparse feature-based stereo rectification, nonlocal-based optical flow estimation for dense matching and finally depth estimation. Employing the proposed approach, three-dimensional (3D) reconstructions of highly complex microscopic samples were achieved to facilitate the interpretation of topology and geometry of surface/shape attributes of the samples. As a byproduct of the proposed approach, high-definition 3D printed models of the samples can be generated as a tangible means of physical understanding. Extensive comparisons with the state-of-the-art reveal the strength and superiority of the proposed method in uncovering the details of the highly complex microscopic samples.

## Introduction

Scanning electron microscope (SEM) is one of the principal research and industrial equipment for imaging on the microscopic scale. SEM and its diverse applications have been a very active research area over the recent decades, and scientific studies well covered the use of SEM in broad domains ranging from biomedical applications to materials sciences and nano technologies [1–7]. SEM as an advanced microscopy device produces high quality images of microscopic specimen using a focused beam of electrons which can be then captured by two types of detectors, secondary electron (SE) and back-scattered electron (BSE) detectors, to provide both compositional and/or geometrical information about the microscopic surface [8]. However, SEM micrographs remain 2D while the need for having a more quantitative knowledge of the 3D surface of the microscopic samples is of high importance. Serial section transmission electron microscopy (ssTEM) [9], serial blockface SEM (SBF-SEM) [10, 11] and focused ion beam SEM (FIB-SEM) [12, 13] are among the widely-used volume electron microscopy devices. While many initially developed as means of imaging of the brain tissues, examples of usage for other biological tissues have been reported in the literature [14]. The procedure of imaging using such devices generally involves sectioning of ultra-thin layers of the tissue and then imaging in order to be able to build a full volume model of the tissue. Sectioning is performed manually in ssTEM while the procedure is done automatically in SBF-SEM (using a diamond knife) and FIB-SEM (using focused gallium ion beam). Using such devices it is possible to acquire high-resolution volume scans of the biological samples. However, due the destructive nature of such imaging procedures, the samples cannot be revisited. Image alignment, rotational errors and charging artifacts may compromise interpretation of volume EM data. The remedies can be sough in specific procedures for specimen preparation or pre-processing steps of image registration. Moreover, segmentation of the features of interest for 3D model reconstruction imposes additional challenges for proper interpretation of such data, especially for the problem of surface assessment since very fine details can be eliminated due to various sources of error mentioned above. These limitations make volume EM imaging not suitable for accurate surface reconstruction of microscopic samples.

The vast literature of used techniques for the problem of surface reconstruction can be categorized into three major classes: a) single-view, b) multi-view, and c) hybrid strategies [15]. In single-view approaches, using varying lighting (electron beam) directions on a single perspective, a group of 2D SEM micrographs are captured and utilized for 3D SEM surface modeling. In multi-view strategies, on the other hand, a set of 2D SEM images from different perspectives assists the 3D SEM surface reconstruction process. The hybrid mechanisms try to combine single-view and multi-view algorithms to restore a 3D shape model from 2D SEM images.

The use of single-view algorithms and the applications to 3D SEM surface reconstruction have been well studied in the literature. The Photometric Stereo (PS) [16] as the major strategy in this class tries to estimate the surface normals of the microscopic sample by observing the object being illuminated from different directions. Paluszynski and Slowko [17] designed a single-view 3D surface modeling approach based on the PS algorithm which also incorporates advanced signal processing algorithms along with both SE and BSE detectors to restore the 3D shape model of SEM images. Pintus et al. [18] developed an automatic alignment strategy for a four-source PS technique for reconstructing the depth map of SEM specimen. Kodama et al. [19] designed a genetic algorithm to tackle the topographical surface reconstruction problem of SEM based on PS method. The proposed genetic algorithm has been applied to the line profile reconstruction from the signals captured by both SE and BSE detectors. Vynnyk et al. [20] proposed a PS based algorithm to 3D SEM surface reconstruction and studied the efficiency of SEM detector system towards a 3D modeling. Slowko and Krysztof [21] designed a PS-based algorithm to reconstruct the 3D surface model of SEM micrographs with the use of angular distribution of back-scattered electron emission to achieve a digital map of surface elevations. This contribution examined different SEM environmental conditions as a high vacuum SEM which was equipped with the BSE detector system utilized for 3D surface reconstruction.

One of the most promising class of methods for 3D surface modeling of SEM images has been the multi-view class which is based on acquisition of multiple images from different perspectives. The Structure from Motion (SfM) [22, 23] and Stereo Vision [24–26] algorithms are advanced visual computational methods which take into account pixels/feature-points matching to assist for accurate 3D SEM surface reconstruction. The class of multi-view 3D reconstruction approaches can be categorized into two major classes: a) sparse feature-based approaches and b) dense pixel based approaches. While methods from the first class are employed to establish a set of robust matches between an image pair or a set of images based on sparsely distributed distinct feature-points, dense multi-view techniques try to discover matches for all points in the images. These matches along with other computational methods will then be used to accurately estimate the projective geometry and 3D surface models [27]. Raspanti et al. [28] presented a high resolution dense multi-view method for 3D reconstructions of biological samples obtained by a SEM. The work implemented novel solutions including a neural adaptive points matching technique to tackle the problem of dense 3D reconstruction. Samak et al. [29] developed a SfM-based algorithm to restore 3D surface model of SEM micrographs. The proposed method initialized a set of 3D points from 2D corresponding points and then triangulated the obtained 3D points into the 3D surface mesh with a mapped texture on the shape model. Carli et al. [30] evaluated the uncertainty of stereo vision algorithm for the problem of 3D SEM surface modeling. Uncertainty for different cases of tilt and rotation were discussed in the work and relative uncertainties of 5% and 4% were achieved for the cases of rotation and tilt, respectively. Zolotukhin et al. [31] studied the pros and cons of SfM algorithm focusing on two-view 3D SEM surface reconstruction problem. Tafti et al. [15] reviewed the state-of-the-art 3D SEM surface reconstruction solutions, addressing several enhancements for the research study, and developed a sparse multi-view algorithm to tackle 3D SEM surface modeling problem. Using machine learning solutions and adaptive strategies, Tafti et al. [32] proposed an improved sparse feature-based multi-view method which outperforms their earlier work in terms of accuracy and computation demands. SEM as an advanced imaging equipment requires careful modification/configuration of internal parameters for 3D reconstruction solutions. Marinello et al. [33] analyzed and studied the 3D reconstruction of SEM images based on different instrumental configurations including calibration, tilt-angle, magnification and etc. Applications of such sparse/dense matching based techniques can also be found in [7, 34], [35] and [36]. Inspired by the above-mentioned approaches, attempts in devising hybrid approaches to combine single-view and multi-view algorithms for restoring the 3D shape model of a microscopic sample have been made too [37].

In single-view 3D surface reconstruction, creating a full model of the microscopic sample is not possible since the images are limited to only one view-point. Moreover, recreating the SEM micrographs of the sample under different illumination conditions is difficult. On the other hand, multi-view approaches offer a more general and achievable framework for the task. However, use of sparse-feature based approach results in blurred edges and smoothed surfaces. This is especially problematic for the very complex microscopic samples, similar to the ones considered here. This requires more advanced matching techniques to capture the very fine details which are missed otherwise, when using sparse feature-based approaches. With the advent of new computer vision-based matching techniques, more accurate and robust approaches can be developed for the problem of 3D surface reconstruction of microscopic samples. In this work, a novel methodology is introduced for high quality 3D reconstruction of microscopic samples using multi-view SEM images. This is to address the growing demand for more accurate reconstruction techniques in fields like biology where the level of complexity of samples is very high. Using the proposed approach, high quality surface meshes of highly complex microscopic samples can be generated which can be used for further quantitative analysis of the surface/shape attributes. The contributions of the current work can be summarized as follows:

1. The current work introduces and investigates a new optimized and robust approach for dense matching and high quality reconstruction of highly complex microscopic samples from sets of multi-view SEM micrographs. Here, a complete framework is proposed in a step-by-step fashion; from image acquisition to pre-processing to dense matching to depth estimation and finally mesh processing and 3D printing.
2. Taking advantage of non-local nature of median filtering, higher accuracy in finding dense matching points are achieved which results in a more truthful reconstruction of 3D surface. Moreover, additional step of weighted median filtering by use of the corresponding micrographs as guidance is proven to reduce the blurring effects near edges and boundaries of the objects.
3. Having a physical model can be beneficial in order to achieve a more realistic representation of the microscopic samples. Therefore, 3D printing of the reconstructed 3D models are considered here. This is to showcase the superior performance of the proposed method in recovering very fine details as well as to provide the means for better understanding of the morphology of the samples.

The rest of the paper is organized as follows. Section *Materials and Methods* contains detailed explanations of the techniques proposed in this work. It covers the SEM imaging protocol used here. After discussing the pre-processing steps of sparse scale invariant feature transform (SIFT) and epipolar stereo rectification, the method of optical flow estimation with non-local regularization is introduced. As for post-processing of the dense matching results, image guided weighted median filtering is introduced next. The section is concluded by true depth estimation using the filtered dense matching results. In Section *Results and Discussions*, the results generated by the proposed framework are presented with detailed comparisons with the state-of-the-art. Section *Conclusion* concludes the paper.

## Materials and methods

### SEM imaging protocol

**Fig 1:**
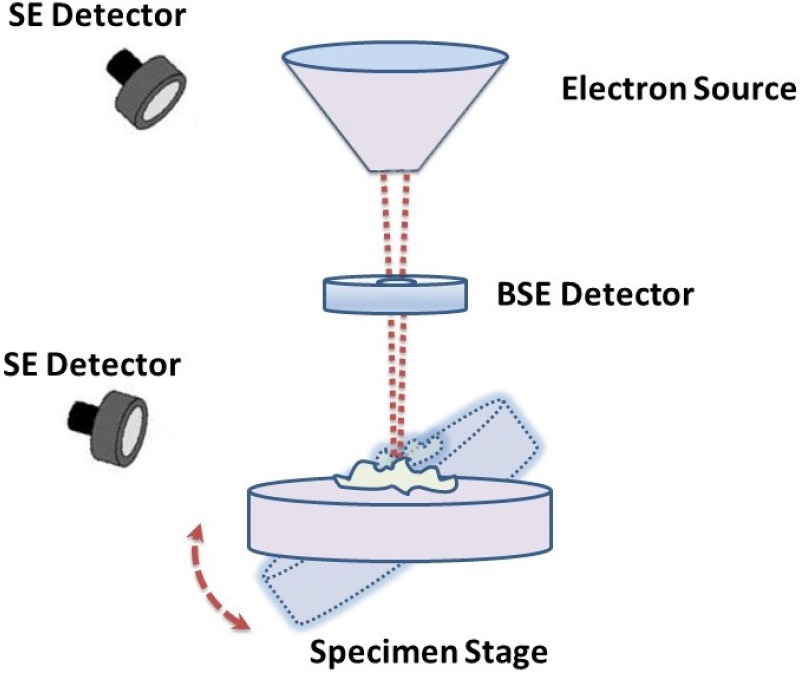
SEM imaging procedure used for this study.

**Fig 2:**
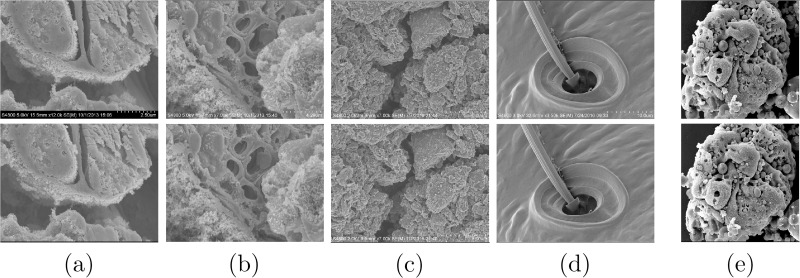
Dataset acquired using a Hitachi S-4800 Field Emission Scanning Electron Microscope (FE-SEM) by tilting the specimen stage by 7°. The samples are (a) *Arabidopsis Anther 1* (1280 × 960), (b) *Arabidopsis Anther 2* (1280 × 960), (c) *Graphene* (1280 × 960), (d) *Pseudoscorpion* (960 × 1280) and (e) *Fly Ash* (926 × 924). The micrographs for the *Pseudoscorpion* set are rotated by 90° for visualization purposes.

In this work, a Hitachi S-4800 field emission scanning electron microscope (FE-SEM) has been utilized to generate the micrographs. This SEM is equipped with a computer controlled 5 axis motorized specimen stage which enables movements in *x*, *y* and *z* directions as well as tilt (-5 to 70°) and rotation (0 to 360°). Specimen manipulations, such as tilt, *z*-positioning and rotation of the specimen stage, as well as image pre-processing and capture functions were operated through the Hitachi PC-SEM software. The working distance which gives the required depth of focus was determined at the maximum tilt for every single sample at the magnification chosen for image capture. As the specimen was tilted in successive 1° increments until reaching the final value through the software application, the SEM image was centered by moving the stage in the *x*- and/or *y*-axes manually. The micrographs were acquired with an accelerating voltage of 3 or 5 kV, utilizing the signals from both the upper and lower SE detectors in a mixed manner, as shown in Fig 1. The magnification and working distance were held fixed in each captured image of the tilt series. Contrast and brightness were adjusted manually to keep consistency between SEM micrographs. Fig 2 summarizes the data that used in this work. Micrographs from *Arabidopsis Anther 1*, *Arabidopsis Anther 2*, *Graphene*, *Pseudoscorpion* and *Fly Ash* are considered for evaluating the performance and accuracy of the proposed approach.

### SIFT feature detection/matching and epipolar rectification

Briefly speaking, four stages of feature detection/description involved in SIFT method can be summarized as [38]: 1) scale-space extrema detection, 2) keypoint localization, 3) orientation assignment and 4) keypoint descriptors. For the first step, a Gaussian function is considered as the scale-space kernel. The local extrema of the response of the image to the difference-of-Gaussian (DoG) masks of different scales is found in a 3 × 3 × 3 neighborhood of the interest point. After several stages of processing for removing the poorly defined keypoints in low contrast regions and near edges using quadratic function fitting and thresholding, the corresponding orientations can be assigned to the keypoints. This is followed by creating a 36-bin histogram for orientations in the keypoint’s neighborhood by considering contributions from each neighbor, weighted based on their gradient magnitude and also by a Gaussian-weighted circular window around the keypoint. Using the location, scale and orientation determined for each keypoint up until now, the keypoint’s descriptor is computed by combining the gradients at keypoint locations, as computed in the previous steps, weighted by a Gaussian function over each 4 × 4 sub-region in a 16 × 16 neighborhood around the keypoint into 8-bin histograms. This results in a 4 × 4 × 8 = 128 element vector for each keypoint.

Given a set of two SEM images of a microscopic sample captured by tilting the specimen stage, the epipolar rectification step aims to transform the images to only have horizontal displacements (disparity) between the corresponding pixels within the images. Assuming a set of sparse naively-matched (using nearest neighbors (NN) algorithm) SIFT feature points followed by *a contrario* RANSAC (ORSA) outlier removal algorithm [39] and represented as 3-vectors of homogeneous coordinates for the left (*X*_*l*_) and right (*X*_*r*_) images, the epipolar constraint can be written as [27]:

$$\begin{array}{c} X_{l}^{T}FX_{r}=0 \end{array}$$ (1)

where F is the fundamental matrix that captures the rigidity constraint of the scene. Having a rectified pair, the fundamental matrix takes the especial form of:

$$\begin{array}{c} F={\left[e_{1}\right]}_{\times }=\left[\begin{array}{ccc} 0 & 0 & 0\\ 0 & 0 & -1\\ 0 & 1 & 0 \end{array}\right] \end{array}$$ (2)

which means that the epipoles are at infinity in horizontal direction. Therefore, the process of rectification involves finding homographies to be applied to the left and right images to satisfy the epipolar constraint equation when *F* = [*e*_1_]_×_:

$$\begin{array}{c} X_{l}^{T}FX_{r}=0\equiv {\left(H_{l}X_{l}\right)}^{T}{\left[e_{1}\right]}_{\times }\left(H_{r}X_{r}\right)=0 \end{array}$$ (3)

Having a rotation matrix *R* for the camera around the focus point, a homography matrix can be formulated as *H* = *KRK*^−1^ where *K* is the camera parameters matrix with (*x*_*c*_, *y*_*c*_) as the image center (principal point) and *f* as the unknown focal length: *K* = [*f* 0 *x*_*c*_;0 *f* *y*_*c*_;0 0 1]. Following the formulation proposed in [40, 41] we look for rotation matrices *R*_*l*_ and *R*_*r*_ and focal length which satisfy:

$$\begin{array}{c} E\left(x_{l},y_{l},x_{r},y_{r}\right)=X_{l}^{T}K^{-T}R_{l}^{T}K^{T}{\left[e_{1}\right]}_{\times }KR_{r}K^{-1}X_{r}=0 \end{array}$$ (4)

where *R*_*r*_ = *R*_*z*_(*θ*_*rz*_)*R*_*y*_(*θ*_*ry*_)*R*_*x*_(*θ*_*rx*_), *R*_*l*_ = *R*_*z*_(*θ*_*lz*_)*R*_*y*_(*θ*_*ly*_) and *K* = *K*(*f* = 3^*g*^(*w*+*h*)), with *w* and *h* as the width and height of the input images respectively and *g* in the range [−1,1]. It should also be noted that due to the specific form of [*e*_1_]_×_ all of the rotations around the *x* direction are eliminated since $R_{x}^{t}{\left[e_{1}\right]}_{\times }R_{x}={\left[e_{1}\right]}_{\times }$. Assuming the Sampson’s error as:

$$\begin{array}{c} E_{s}^{2}=E^{T}{\left(JJ^{T}\right)}^{-1}E \end{array}$$ (5)

where *J* is the matrix of partial derivatives of *E* with respect to the 4 variables:

$$\begin{array}{c} J=({\left(FX_{r}\right)}_{1}\;\;{\left(FX_{r}\right)}_{2}\;\;{\left(F^{T}X_{l}\right)}_{1}\;\;{\left(F^{T}X_{l}\right)}_{2}) \end{array}$$ (6)

we have

$$\begin{array}{c} E_{s}{\left(X_{l},X_{r}\right)}^{2}=\frac{E{\left(X_{l},X_{r}\right)}^{2}}{\left|\right|{\left[e_{3}\right]}_{\times }F^{T}X_{l}{\left|\right|}^{2}+\left|\right|{\left[e_{3}\right]}_{\times }FX_{r}{\left|\right|}^{2}} \end{array}$$ (7)

Utilizing Levenberg-Marquardt [42], the method seeks the parameters (*θ*_*ly*_, *θ*_*lz*_, *θ*_*rx*_, *θ*_*ry*_, *θ*_*rz*_, *g*) which minimize the sum of Sampson errors over the matching pairs. The optimized parameters are then used for building the two homographies to be applied to the left and right view images. More elaboration regarding the theory and implementation aspects of the rectification method can be found in [40, 41].

### Dense matching by optical flow estimation

Finding a dense matching map between individual pixels of the input SEM micrographs is of high importance for high quality depth estimation and point cloud generation. One should note that the images are captured of rigid objects, with the only change being in the viewpoint angle. The rigidity of the microscopic samples, then, should be preserved in the found dense correspondence maps. This is generally satisfied since the imaged objects are well-textured which makes the process of matching more robust. On the other hand, edges/discontinuities contained in the micrographs should be preserved. This is mainly necessary for distinguishing different regions of more complex microscopic samples, similar to that of considered here (refer to Fig 2). Being able to preserve the discontinuities benefits the depth estimation greatly. However, the found correspondence maps should be piece-wise smooth which is usually satisfied in the formulation of energy functional required for matching. For the current work, dense matching is achieved using high quality optical flow estimation.

Optical flow estimation introduced by [43] refers to the estimation of displacements of intensity patterns in image sequences [44], [45]. Generally speaking, the problem can be formulated as a global energy optimization problem of the form *E*_*Global*_ = *E*_*Data*_+*λE*_*Prior*_ where the data term, *E*_*Data*_, measures the consistency of the optical flow for the input images and the prior term, *E*_*Prior*_, applies additional constraints for having a specific property for the flow field, for example smoothly varying flow fields. The choice of each term in the global energy functional and also the optimization algorithms varies in different methods for optical flow estimation. Assuming a two-frame (*I*_1_ and *I*_2_) formulation, the objective function can be written as:

$$\begin{array}{c} \begin{array}{cc} E(u,v)= & \sum_{i,j}\{p_{D}\left(I_{1}\left(i,j\right)-I_{2}\left(i+u_{i,j},j+v_{i,j}\right)\right)\\ & +\lambda [p_{S}\left(u_{i,j}-u_{i+1,j}\right)+p_{S}\left(u_{i,j}-u_{i,j+1}\right)\\ & +p_{S}\left(v_{i,j}-v_{i+1,j}\right)+p_{S}\left(v_{i,j}-v_{i,j+1}\right)]\} \end{array} \end{array}$$ (8)

with **u** and **v** as the horizontal and vertical components of the flow field, *i*, *j* as the pixel indexes, *λ* as the regularization parameter and finally, *p*_*D*_ and *p*_*S*_ as the data and spatial prior penalty functions, respectively. In the original work of Horn and Schunck [43] quadratic functions are used for both the data and spatial penalty functions. But in the literature examples of using Charbonnier $\left(p\left(x\right)=\sqrt[{}]{x^{2}+{∊}^{2}})\right)$ in [46] and Lorentzian $\left(p\left(x\right)=\text{log}\left(1+\frac{x^{2}}{2\sigma^{2}}\right)\right)$ in [47] penalty functions and their variants can be found which provide a more robust estimation of the underlying flow fields. To account for large displacements between frames, the above formulation is usually minimized in a multi-resolution manner using incremental pyramid schemes, with steps of Gaussian anti-aliasing and flow outlier removal filters between iterations. This helps the process of linearization of the objective function manageable and ensures lower chances of being trapped in local optima. As thoroughly discussed in [48], however, median filtering of the optical flow estimates after each pyramid level has a big impact in the final outcome of the minimization process: while the final energy is higher than what is achieved without median filtering, the optical flow error is minimized. This is due to the *non-local* nature of median filtering which is different from the local pairwise smoothness term. Incorporating the non-local median filtering heuristic as a weighted term in the energy functional can be considered as a means for ensuring minimal over-smoothing across boundaries. This is empirically useful for the problem of dense matching in SEM stereo pairs, especially since very fine details has to be preserved to obtain a more accurate 3D reconstruction.

Explicit formulation of the median filtering in Eq 8 can be approximated by

$$\begin{array}{c} \begin{array}{cc} E(u,v)= & \sum_{i,j}\{p_{D}\left(I_{1}\left(i,j\right)-I_{2}\left(i+u_{i,j},j+v_{i,j}\right)\right)\\ & +\lambda [p_{S}\left(u_{i,j}-u_{i+1,j}\right)+p_{S}\left(u_{i,j}-u_{i,j+1}\right)\\ & +p_{S}\left(v_{i,j}-v_{i+1,j}\right)+p_{S}\left(v_{i,j}-v_{i,j+1}\right)]\}\\ & +\lambda_{N}\sum_{i,j}\sum_{\left(i^{\prime },j^{\prime }\right)\in N_{i,j}}\left(\right|u_{i,j}-u_{i^{\prime },j^{\prime }}\left|+\right|v_{i,j}-v_{i^{\prime },j^{\prime }}\left|\right) \end{array} \end{array}$$ (9)

in which $N_{i,j}$ is the neighborhood centered at (*i*, *j*) and *λ*_*N*_ is the weight determining the contribution of the non-local weighted median term. Due to difficulty of optimization of Eq 9 when having large spatial terms, the objective function can be relaxed using a set of auxiliary horizontal ($\hat{u}$) and vertical ($\hat{v}$) flow field components:

$$\begin{array}{c} \begin{array}{cc} E(u,v,\hat{u},\hat{v})= & \sum_{i,j}\{p_{D}\left(I_{1}\left(i,j\right)-I_{2}\left(i+u_{i,j},j+v_{i,j}\right)\right)\\ & +\lambda [p_{S}\left(u_{i,j}-u_{i+1,j}\right)+p_{S}\left(u_{i,j}-u_{i,j+1}\right)\\ & +p_{S}\left(v_{i,j}-v_{i+1,j}\right)+p_{S}\left(v_{i,j}-v_{i,j+1}\right)]\}\\ & +\lambda_{C}\left(|\right|u-\hat{u}{\left|\right|}^{2}+\left|\right|v-\hat{v}{\left|\right|}^{2})\\ & +\lambda_{N}\sum_{i,j}\sum_{\left(i^{\prime },j^{\prime }\right)\in N_{i,j}}\left(\right|{\hat{u}}_{i,j}-{\hat{u}}_{i^{\prime },j^{\prime }}\left|+\right|{\hat{v}}_{i,j}-{\hat{v}}_{i^{\prime },j^{\prime }}\left|\right) \end{array} \end{array}$$ (10)

where *λ*_*C*_ is a scalar weight which penalizes the contribution of differences between the auxiliary and main flow fields. The current formulation with *L*_1_ minimization is in close accordance with median filtering [49]. Assuming the above explicit representation of median filtering as part of the energy minimization functional, more improvement can be achieved by employing a weighted approach based on the approximate classification of the pixels in the neighborhood. In the non-local term, given a pixel and knowing which pixels in the neighborhood belong to the same surface, higher weights can be assigned while for the other pixels weights are lower [50]. In this manner, the non-local term in Eq 10 is replaced with:

$$\begin{array}{c} \sum_{i,j}\sum_{\left(i^{\prime },j^{\prime }\right)\in N_{i,j}}w_{i,j}^{i^{\prime },j^{\prime }}\left(\right|{\hat{u}}_{i,j}-{\hat{u}}_{i^{\prime },j^{\prime }}\left|+\right|{\hat{v}}_{i,j}-{\hat{v}}_{i^{\prime },j^{\prime }}\left|\right) \end{array}$$ (11)

The weights $w_{i,j}^{i^{\prime },j^{\prime }}$ can be approximated by taking the spatial distance, color-value distance and occlusion states into account [51–53]:

$$\begin{array}{c} w_{i,j}^{i^{\prime },j^{\prime }}\propto \exp\left\{-\frac{|i-i^{\prime }{|}^{2}+{\left|j-j\right|}^{2}}{2\sigma_{1}^{2}}-\frac{|I\left(i,j\right)-I\left(i^{\prime },j^{\prime }\right){|}^{2}}{2\sigma_{2}^{2}n_{c}}\right\}\frac{o(i^{\prime },j^{\prime })}{o(i,j)} \end{array}$$ (12)

where **I** is the color vector in the Lab color space, *n*_*c*_ is the number of color channels, *σ*_1_ = 7 and *σ*_2_ = 7. The occlusion variable *o*(*i*, *j*) is defined as:

$$\begin{array}{c} o\left(i,j\right)=\exp\left\{-\frac{d^{2}\left(i,j\right)}{2\sigma_{d}^{2}}-\frac{{\left(I\left(i,j\right)-I\left(i+u_{i,j},j+v_{i,j}\right)\right)}^{2}}{2\sigma_{e}^{2}}\right\} \end{array}$$ (13)

where *d*(*i*, *j*) is the one-sided divergence function (only negative values, and positives considered as zero). This variable is near zero for occluded pixels while close to one in non-occluded regions. The parameters *σ*_*d*_ and *σ*_*e*_ are set to 0.3 and 20, respectively according to [51]. Following the work of Li and Osher [54], an approximate solution for the auxiliary flow filed components, $\hat{u}$ and $\hat{v}$, can be found for all of the pixels.

Full implementation of the above requires high computational power. A simple modification can reduce the computational need immensely. Since the weighted formulation is designed to overcome the negative impacts of over-smoothing boundaries in the process of optical flow estimation while the estimates in the uniform regions are very accurate, different methodologies can be applied to ensure an accurate solution while demanding less computational power. Using a Sobel edge detector and having the current estimate of optical flow, motion boundaries can be detected and then dilated to determine the flow boundary regions. In these regions the weighted formulation with a 15 × 15 neighborhood is applied while in non-boundary regions, a 5 × 5 un-weighted approach is taken. This will reduce the computational time drastically.

Optimizing Eq 10 will result in the flow field representing how the pixels moved between the micrographs. Given that the input micrographs are rectified, the vertical components of the flow fields are negligible in comparison to the horizontal components. In fact, the energy of the vertical disparity map is less than 1% of that of horizontal disparity. Considering this, the vertical disparity map is disregarded for the rest of the steps.

### Disparity refinement by weighted median filter

As can be seen from the micrographs used in the current work, the level of detail can be very high due to presence of many microscopic objects in the samples. This can be mainly problematic since the variation of the size is also large. A great representative is the *Fly Ash* sample in which objects of various sizes as well as regions with different textures are present. This cannot be fully recovered by the previous steps and therefore, our goal of a more truthful 3D reconstruction can be compromised. However, this can be greatly remedied by using the original images for guiding towards a more accurate correspondence. Here, we propose to use *weighted median filtering* as means for error correction. In this manner, the original images serve as guidance for a more accurate filtering of the computed disparity map.

Weighted median filter, as is obvious from the name, aims to replace the image pixels with weighted median of the neighborhood pixels within a local window [55, 56]. Assuming image *I* and the corresponding feature map **f**, and pixel *p* in image *I* located at the center of a local window R(p) with radius *r*, for each pixel q∈R(p) a weight *w*_*pq*_ will be assigned which is a representative of the affinity of the two pixels in the feature map **f**. This can be represented as

$$\begin{array}{c} w_{pq}=g\left(f\left(p\right),f\left(q\right)\right) \end{array}$$ (14)

where *g* is the influence function (Gaussian, reciprocal, cosine, etc.) [57]. Given *n* = (2*r*+1)^2^ as the number of pixels in the local window R(p), the value and weight element of all *n* pixels can be expressed as {(*I*(*q*), *w*_*pq*_)}. After sorting values in an ascending order, the weighted median operator returns the new pixel p* such as:

$$\begin{array}{c} p^{*}=min\;k\;s.t.\;\sum_{q=1}^{k}w_{pq}\geq \frac{1}{2}\sum_{q=1}^{n}w_{pq}. \end{array}$$ (15)

which means that the sum of corresponding weights for all pixels before p* should be almost half the sum of all weights. It should be noted that in this formulation, feature map **f** determines the weights.

For our work, use of weighted median filtering is considered for achieving a more accurate correspondence. Given the computed disparity map from the previous step, and also having the first micrograph from each image set that is used for optical flow estimation as the feature map, the disparity map is filtered using the weighted median filter. Even though the straightforward implementation of the method is simple, it can be very time consuming due to spatially varying wights and the median property. Zhang et al. [57] proposed the use of joint-histogram with median tracking and necklace table data structure for fast implementation of the weighted median filter. Employing this approach, a more detailed disparity map can be achieved which results in a higher fidelity 3D reconstruction.

### Depth estimation

Stereo rectification transforms the images in a manner in which the displacements will be grossly concentrated in the horizontal direction. This greatly simplifies the process of depth estimation. This is especially useful for the case of 3D reconstruction of SEM images since the tilt angles are very small with high amount of overlap between stereo image pairs. For more general problems like large scale multiple view stereo (MVS), the proposed technique is not directly applicable and more sophisticated methods are needed [58–60].

**Fig 3:**
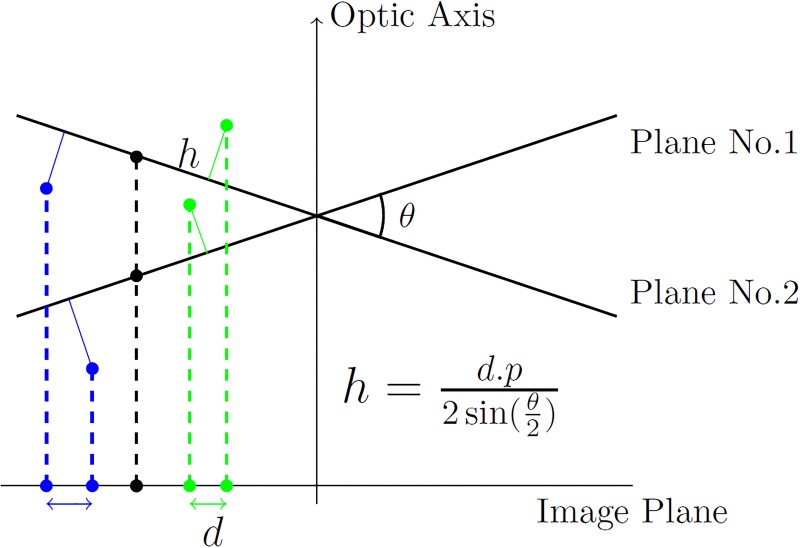
Relationship between the estimated height (*h*) and the computed horizontal disparity (*d*) using the pixel size in sample units (*p*) and the total tilt angle (*θ*).

The horizontal disparity computed from the previous step, can be utilized for estimating the depth of the individual pixels contained in the images. This requires several parameters to be known: tilt angle, magnification factor and size of each pixel in sample units. Fig 3 shows the relationship between the computed horizontal disparity and the height for a few sample points. This can be represented using a simple trigonometric equation [61–63]:

$$\begin{array}{c} h=\frac{d.p}{2\sin(\frac{\theta }{2})} \end{array}$$ (16)

which uses the computed horizontal disparity *d*, pixel size in sample units (*p*) and the total tilt angle (*θ*) to estimate the height (*h*).

## Results and discussions

Assessing the performance of the proposed method is done in several steps both qualitatively and quantitatively. Using a Hitachi S-4800 field emission scanning electron microscope (FE-SEM), the micrographs of the five sample sets (*Arabidopsis Anther 1*, *Arabidopsis Anther 2*, *Graphene*, *Pseudoscorpion* and *Fly Ash*) are captured. The device is equipped with computer controlled 5 axis specimen stage which enables movements along three coordinate axis as well as tilting and rotation. The process of image acquisition is done in a controlled manner by an expert with manual adjustments of focus and re-centering when needed. Between the two views acquired for each sample, only the tilt angle is changed while the distance between the specimen stage and the detectors as well as zooming factor are kept constant. In order to keep the image acquisition sessions consistent, the tilt angle between micrographs of each set is set to 7°. However, similar tilt angles in close range would produce the same results as evidenced by our previous experiments. One should note that the amount of overlap between images is a key factor in a more accurate 3D reconstruction. Keeping the tilt angle small, as well as re-centering the sample after tilting the specimen stage will ensure a more accurate and robust matching and therefore result in a more truthful reconstruction.

The first step of the proposed approach consists of finding distinctive feature points in the two input micrographs from each set to be used for stereo rectification. Given the initial SIFT feature points, SIFT descriptors are computed as described in Section *SIFT Feature Detection/Matching and Epipolar Rectification*. This is followed by putative matching of the SIFT descriptors considering naive nearest neighbor search. Since it is assumed that SIFT descriptors capture information about the neighborhood of each feature point, putative matching produces reasonable number of correct matches. However, it cannot be expected to have a completely accurate matching between feature points due to noise and also similarities in textures contained in the input images. Therefore, one should find a reasonable transform between the matching points that satisfies some error criteria for the majority of matched features. In our work, without going into much detail as this subject is a very well-studied concept in computer vision, a variant of random sample consensus (RANSAC), namely *a contrario* RANSAC (ORSA), is used in order to find correct matches that satisfy a homography transform between the two images. This is followed by formulating the Sampson’s error to be used for rectifying the input pair in order to have horizontally concentrated matchings. This step is necessary for the process of dense matching needed for high quality 3D reconstruction. In sparse feature-based approaches used for 3D reconstruction of microscopic samples [32, 64], computation of fundamental matrix and the subsequent projective transformation is computationally efficient. This is due to small number of matching points in comparison to the total number of pixels in the images. However, having the dense matching for all the pixels in the images requires specific configurations. Rectifying the input pair simplifies the problem of 3D point cloud generation. In this case, the need for computing the fundamental matrix and projective transformation using all of the matching points is eliminated. Table 1 summarizes the result of the rectification process used for this study for all of the sample sets. The first and second row in the table represents the number of individual SIFT feature points found in the input images. This is followed in the third row by the number of true matches after putative nearest neighbor matching and ORSA outlier elimination. Even though this number consists of a small portion of the initial matches, however, for the purpose of stereo rectification is enough. The number of initial and final matches is lower for the *Pseudoscorpion* set due to lower amount of variations and texture in the images of the set. The table continues with the initial and final rectifications errors obtained using the quasi-Euclidean stereo rectification process. Having a more horizontally-concentrated matching between image pixels will ensure more accurate and robust 3D reconstruction.

**Table 1:** Rectification results: number of SIFT points found in each input image (rows 1 and 2), number of matching points after *a contrario* RANSAC (row 3), initial and final rectification errors from before and after the quasi-Euclidean rectification (rows 4 and 5). As can be seen, despite careful image acquisition, the initial rectification errors are large.

	*Arabidopsis Anther 1*	*Arabidopsis Anther 2*	*Graphene*	*Pseudoscorpion*	*Fly Ash*
im.1 # SIFT keypoints	783	981	2089	195	1633
im.2 # SIFT keypoints	658	893	2488	65	1652
ORSA # SIFT matches	**214**	**268**	**487**	**18**	**418**
initial rect. err. (pix)	2.393	6.910	14.055	1.223	2.766
final rect. err. (pix)	**0.802**	**0.425**	**0.277**	**0.971**	**0.472**

**Fig 4:**
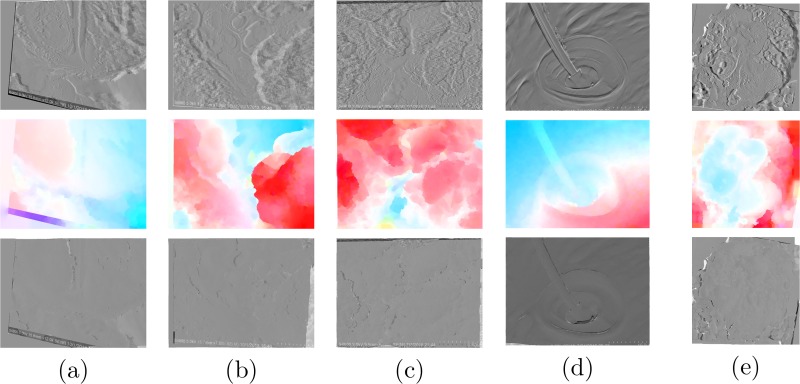
Optical flow estimation results for (a) *Arabidopsis Anther 1*, (b) *Arabidopsis Anther 2*, (c) *Graphene*, (d) *Pseudoscorpion* and (e) *Fly Ash* sample sets. The first row shows the initial difference maps. The second row shows the computed optical flow estimate. Using the optical flow estimate, the first image in each pair is warped and then used for generating the final difference maps as depicted in the third row. It should be noted that the images for *Pseudoscorpion* set are rotated by 90° for visualization purposes.

The rectification step is followed by optical flow estimation to determine the dense matching between individual image pixels in the image pair. Fig 4 shows the results of optical flow estimation. For better visualization of the effects of dense matching, the difference maps are displayed. The first row shows the initial difference map between the input images of the pair. The second row shows the estimated optical flows for compensating the movements of individual pixels in the two images. The computed flow is color-coded, with red representing positive values and blue representing negative values. Utilizing the computed optical flow estimates, the first image of the pair can be warped to generate the second image. The difference maps between the warped first image and the second image of each pair are excellent representatives of the performance of the matching procedure. These are shown in the third row of Fig 4. Inspecting the computed optical flows reveals very important properties of the image matching that is required here. Two dimensional deformable biomedical image registration as an example of image matching tries to find the correspondence between pixels of two images [65, 66]. However, the general formulation assumes that the matching points are all laid on the same plane. This is not the case for many computer vision problems, optical flow estimation included. In such cases the computed correspondence must be discontinuity preserving. In other words, an image as a projective depiction of a scene may contain several objects which are actually lay on the same plane and can move independently and therefore, the computed flow patterns should account for that [67].

Having a rectified stereo pair as input to the optical flow estimation approach results in a horizontally-concentrated flow estimate, as expected. Our experiments show that the energy contained in the vertical component of optical flow is less than 1% of the horizontal component, which is ideal for an accurate reconstruction. Therefore, for 3D reconstruction, only the horizontal component is used as the disparity map.

**Fig 5:**
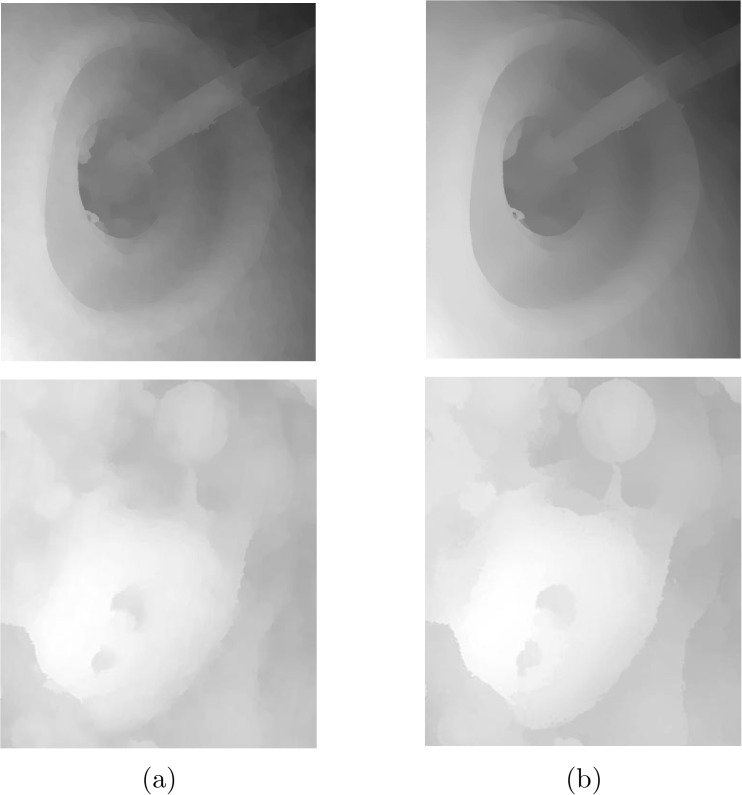
Effects of weighted median filtering on the horizontal disparity map: a) before and b) after. Despite inclusion of non-local term in the optical flow energy functional, the outcome can be improved greatly by adding an additional weighted median filtering step.

Even though the employed optical flow approach produces highly accurate results, due to lack of color in the initial SEM images, the results may suffer from blurred edges. This is mainly problematic in highly complex samples used here, *Fly Ash* for example. To ensure a more accurate estimation, further post-processing is done using weighted median filtering as described in Section *Disparity Refinement by Weighted Median Filter*. Using the first image as guidance, because the optical flow is computed from the first to the second image in the pair, the disparity map is filtered taking advantage of weighted median filtering. Fig 5 shows the effects of the employed post-processing filtering on portions of the *Pseudoscorpion* and *Fly Ash* disparity maps. While the initial disparity maps has blurred edges and bumpy appearances, the result of weighted median filtering is more sharp and accurate near the edges. Moreover, more detail is preserved in the resulted disparity map as can be seen from the presented images.

**Fig 6:**
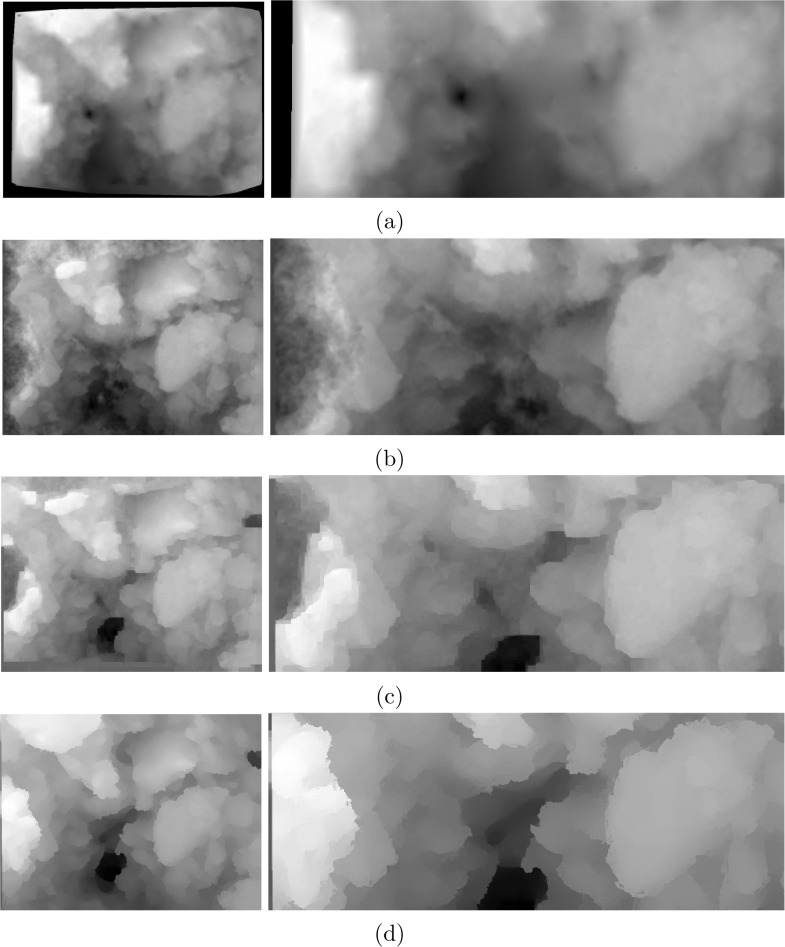
Comparison of the results for *Graphene*: a) the overall as well as a zoomed region of the computed disparity map using the state-of-the-art method of [32] which uses sparse feature-based matching approach and *a contrario* RANSAC for outlier removal. The dense disparity map is created by scattered data interpolation of the sparse disparity values. b) the result of Horn/Schunck optical flow estimation [43], which provides a better estimation of the disparity map than that of [32]. c) the result of dense feature matching proposed in [68] which uses dense SIFT features as well as factor graph representation of the matching energy functional optimized by loopy belief propagation. Even though relatively better than [43], the result still suffers from blurred edges. The result of the proposed method is presented in (d). In comparison to the state-of-the-art, the proposed approach generates a sharper and more accurate disparity map.

**Fig 7:**
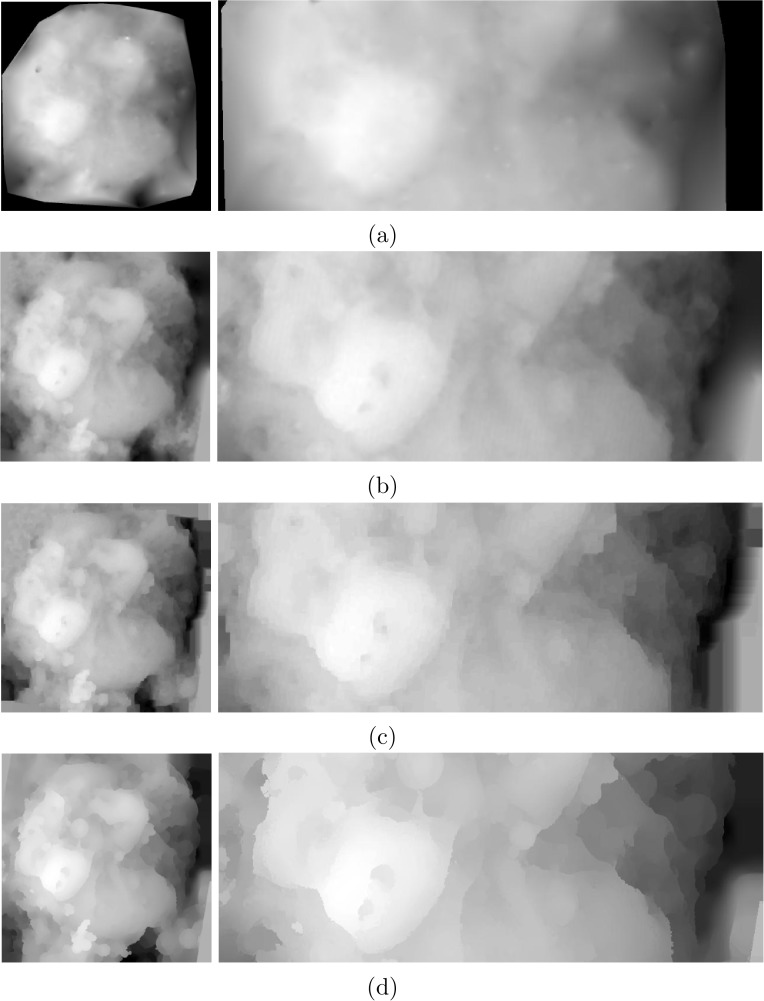
Comparison of the results for *Fly Ash*: a) the overall as well as a zoomed region of the computed disparity map using the state-of-the-art method of [32] which uses sparse feature-based matching approach and *a contrario* RANSAC for outlier removal. The dense disparity map is created by scattered data interpolation of the sparse disparity values. b) the result of Horn/Schunck optical flow estimation [43], which provides a better estimation of the disparity map than that of [32]. c) the result of dense feature matching proposed in [68] which uses dense SIFT features as well as factor graph representation of the matching energy functional optimized by loopy belief propagation. Even though relatively better than [43], the result still suffers from blurred edges. The result of the proposed method is presented in (d). In comparison to the state-of-the-art, the proposed approach generates a sharper and more accurate disparity map.

For a more comprehensive analysis, the proposed dense matching approach is compared with several other methods previously used in the literature for dense matching and subsequently 3D reconstruction. Sparse feature-based approaches track the movements of distinct feature points in the input images in order to compute the fundamental matrix and projective transformation [32, 64]. To generate a dense disparity map, similar to that of created by our approach for a better comparison of the performance, the sparse disparity values are interpolated employing a Delaunay triangulation-based interpolation method. As for dense matching schemes, the works of Horn and Schunck [43] and Liu et al. [68] are good examples. While the first one works based on the pixels’ correspondence, the later extends a similar idea to matching of dense SIFT descriptors. Figs 6 and 7 display the disparity maps computed using the above-mentioned methods as well as the proposed approach for the *Graphene* and *Fly Ash* sample sets, respectively. In each figure, the left column shows the overall disparity map while the right column is a zoomed view for a better visual comparison of the various techniques. Close inspection of the provided results displays the superiority of the proposed approach. As expected, the outcome of the sparse feature-based approach is highly blurred near edges with significant loss of details presented in the images. Even though such techniques are mainly used with more than two input images, the performance is the same as evident from the results. In contrast, dense matching approaches produce more accurate results. In the results of the method of [43], more details are presented and discontinuities are better preserved. However, in cases of having larger displacements near the margins of the input images (left side of the *Graphene* results) the estimated optical flow is not as accurate as the sparse feature-based approach. Using the dense descriptor matching scheme in the work of Liu et al. [68], this is mostly resolved. In this technique, at first two 128-dimensional dense SIFT descriptor images of both the first and second micrograph in the pair are created. To compute the matching, a factor graph representation of the specifically defined energy functional is introduced and the process of optimization is done using loopy belief propagation. By employing the dense descriptor matching methodology more accurate results can be achieved. The last row in Figs 6 and 7 is the disparity results using the proposed approach. Employing the proposed approach, higher levels of details can be reached in the resulted disparity maps. With higher accuracy in preserving the discontinuities, a more truthful reconstruction can be made. This is more evident in the samples with higher complexity levels, *Fly Ash* sample set for example. As shown in Fig 7, the proposed approach can recover disparity values even for smaller objects in the images, while in contrast, the other methods presented here cannot, due to high amount of blur around edges and boundaries.

**Fig 8:**
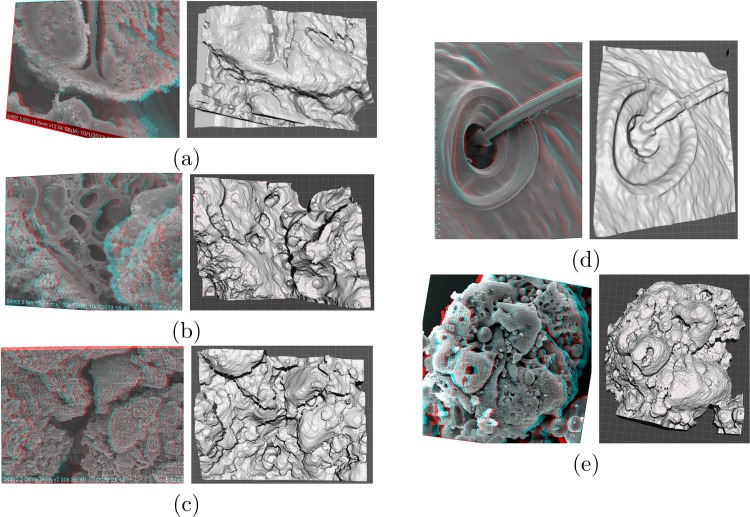
3D red-cyan anaglyphs generated by combining the two rectified stereo views of the microscopic samples as well as the solid 3D models created using Meshmixer [70] for (a) *Arabidopsis Anther 1*, (b) *Arabidopsis Anther 2*, (c) *Graphene*, (d) *Pseudoscorpion* and (e) *Fly Ash* sample sets.

**Fig 9:**
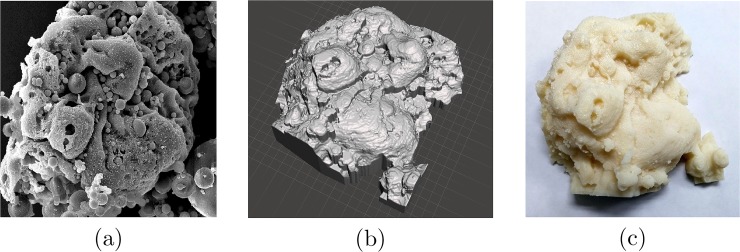
From start to finish: (a) first image from the *Fly Ash* sample set, (b) 3D solid model generated using the computed disparity estimates and modified using MeshMixer [70], (c) 3D printed model. Using the proposed approach, highly complex structure of the sample was captured and reconstructed in the printed model.

Having the height estimate for each point, a dense 3D point cloud can be generated and further used for 3D surface reconstruction. To eliminate the effects of smoothing introduced by general purpose mesh generating toolsets, similar to that of used in MeshLab [69], Delaunay triangulation is done by utilizing the image grid as the set of mesh nodes. The triangular mesh nodes are then transformed from the two dimensions of the image plane to the three dimensions of the model using the computed height estimates. This practically eliminates the smoothing effects which generally happen near the edges of the objects and in areas that contain sudden jumps due to sharp changes in the depth estimate. Using simple MATLAB scripts, the generated 3D surface can be transformed and saved as standard STL files which can be later used for further mesh modification and processing using more specialized software. Use of edge aware mesh smoothing procedures can be considered in order to have a more pleasing appearance without losing details of the edges and sudden changes of depth. Fig 8 shows 3D red-cyan anaglyphs generated by combining the two rectified stereo views of the microscopic samples as well as the solid 3D models created using Meshmixer [70]. The generated models can also be sent out for 3D printing as the ultimate means for creating a tangible representation of the complex microscopic structure [34, 36, 71, 72]. Fig 9 (a) shows one image from *Fly Ash* sample, while (b) shows another view of the 3D solid model created using the computed disparity estimates and modified using MeshMixer. Finally, (c) is an image captured from the 3D printed model.

## Conclusion

In this work, a novel and accurate approach is introduced for high fidelity 3D reconstruction of highly complex microscopic samples. In the proposed methodology, multi-view SEM micrographs from two different view-points are captured using a Hitachi S-4800 field emission scanning electron microscope (FE-SEM). The micrographs are acquired with 7° tilt angle difference, made possible by the provided computer controlled 5 axis specimen stage. The image acquisition is then followed by one stage of pre-processing which consists of four steps: a) sparse SIFT feature detection/description, b) nearest neighbor search for finding the putative sparse matching, c) *a contrario* RANSAC for outlier removal and finally d) quasi-Euclidean stereo rectification. This step is necessary due to the need for high quality dense correspondence required for accurate 3D reconstruction of highly complex samples used here. The pre-processing stage is followed by dense matching, employing non-local based optical flow estimation. Using this technique, a highly accurate estimate of dense correspondence can be achieved. To ensure a more accurate disparity map as well as eliminating blurred edges, a post-processing filtering step using weighted median filtering is done which uses the first image in each pair as the guidance. Finally, the disparity map is used to generate the 3D point cloud of the microscopic sample. The 3D point cloud is later used for high quality surface mesh generation. Extensive comparisons reveal the superiority of the proposed method to the state-of-the-art sparse feature-based techniques used for 3D surface reconstruction of SEM images. Moreover, the produced results are experimentally proven to be extremely accurate and suitable for 3D printing.

The provided results can serve as great qualitative measures for assessing the performance of the proposed method. However, having a more quantitative measurement of the performance is of high importance. The solution to the problem can be sought in direct/indirect measurements of surface/volume properties in conjunction with 3D surface reconstruction. Such properties may include, but not limited to, surface roughness, particle size/volume measurement and coefficient of friction estimation. Imaging modalities such as Atomic Force Microscopy (AFM) [73] can be considered as means of assessment. Moreover, depending on the sample’s properties, volume electron microscopy imaging modalities mentioned before (e.g. ssTEM, SBF-SEM or FIB-SEM) can also be used for generating accurate 3D volume models of the samples. However, it should be noted that the destructive nature of the volume microscopy modalities prevents us from revisiting the samples. Moreover, errors in accurately delineating the features of interest (here, surfaces) may compromise the analysis. More investigations on the subject is left for future research.
